# International Society for Computational Biology Welcomes Its Newest Class of Fellows

**DOI:** 10.1371/journal.pcbi.1003199

**Published:** 2013-08-22

**Authors:** Christiana N. Fogg, Diane E. Kovats

**Affiliations:** 1 Freelance Science Writer, Kensington, Maryland, United States of America; 2 Executive Director, International Society for Computational Biology, La Jolla, California, United States of America

Computational biology and bioinformatics have grown into cutting-edge interdisciplinary fields essential to every realm of basic research in this age of modern genomics and open-source software. In light of this, the International Society for Computational Biology (ISCB) saw a need to estabish a program for the organization that recognized its members who made significant contributions to the fields, as well as service to the Society. A task force led by Mona Singh was formed to develop a program to recognize and honor this unique group of researchers.

In early 2009, ISCB established its Fellows Program. The first group elected into this distinguished group were past recipients of the ISCB Accomplishment by a Senior Scientist Award, which was established in 2003. Since then, Fellows have been identified through a rigorous process involving a call for nominations from the ISCB membership and selection by the Fellows Selection Committee, which in past years has included the ISCB Board of Directors and previously selected Fellows.

The distinction of an ISCB Fellow is based on:

- Nomination by an ISCB member submitting a short endorsement for the nominee, a detailed half-page statement of motivation justifying the nominee, and a CV of the nominee.
- Nominees must have demonstrated excellence in research, service to the ISCB community, education, and/or administration.
- Nominees must have been a member of the Society for at least three of the last six years.

Selection of new Fellows each year is limited to half of one percent of the previous year's membership and includes the Senior Scientist Award winner.

The 2013 ISCB Fellows epitomize the mission of ISCB to advance scientific understanding of living systems through computation and clearly fulfill the goals of the Fellows Program. Each Fellow has made outstanding contributions to computational biology through research, teaching, and service to the scientific community. On behalf of the ISCB Board of Directors and Fellows Committee, congratulations!

## Pierre Baldi

Pierre Baldi (Image 1) is a Chancellor's Professor in the Department of Computer Science and Director of the Institute for Genomics and Bioinformatics at the University of California, Irvine. Baldi holds a Ph.D. in mathematics from the California Institute of Technology, and he is a leader in the fields of artificial intelligence and machine learning. He has used these approaches for comparative genomics, computer-based drug design, and modeling of metabolic, neural, and signaling networks. Baldi's innovative research has been recognized by several awards, including the Lew Allen Research Award and the Microsoft Faculty Research Award. He has published numerous high-impact papers, and he has authored four books that span topics from microarrays, bioinformatics, and data mining.

**Figure:**
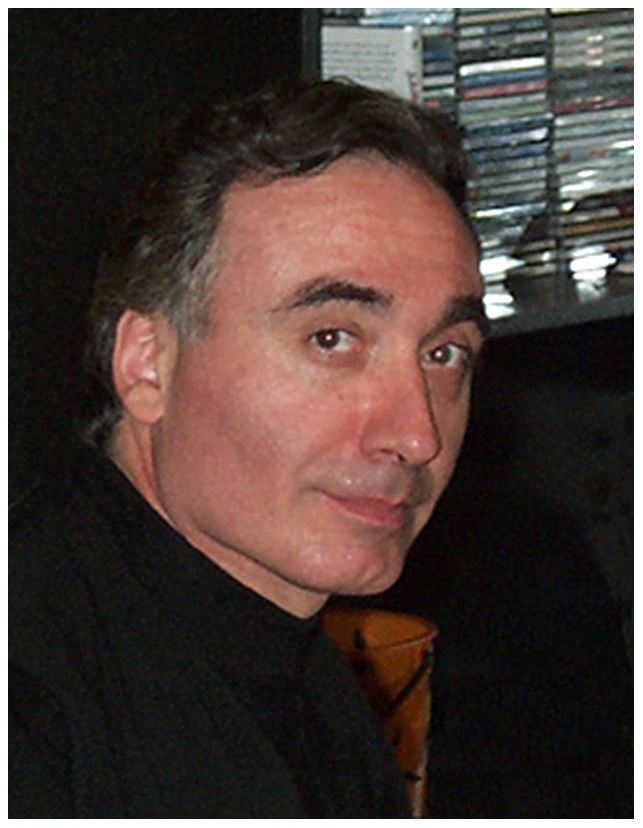
Image 1. Pierre Baldi. Image credit: Pierre Baldi.

## David Eisenberg

David Eisenberg (Image 2) is a Professor in the Departments of Chemistry and Biochemistry, and Biological Chemistry at the University of California, Los Angeles, and he is also a Howard Hughes Medical Institute Investigator. Eisenberg received his D.Phil. in theoretical chemistry from Oxford University. Eisenberg is the winner of the 2013 ISCB Accomplishment by a Senior Scientist Award. Eisenberg has spent several decades elucidating numerous novel protein structures through X-ray crystallography. At present, his research is focused on solving the structures of amyloid-forming proteins, which are associated with several debilitating neurodegenerative diseases. He has also been a pivotal contributor to the development of bioinformatics databases and tools that analyze protein interactions. Eisenberg has published over 300 papers and is the recipient of numerous awards honoring his groundbreaking contributions to the fields of biochemistry and bioinformatics.

**Figure:**
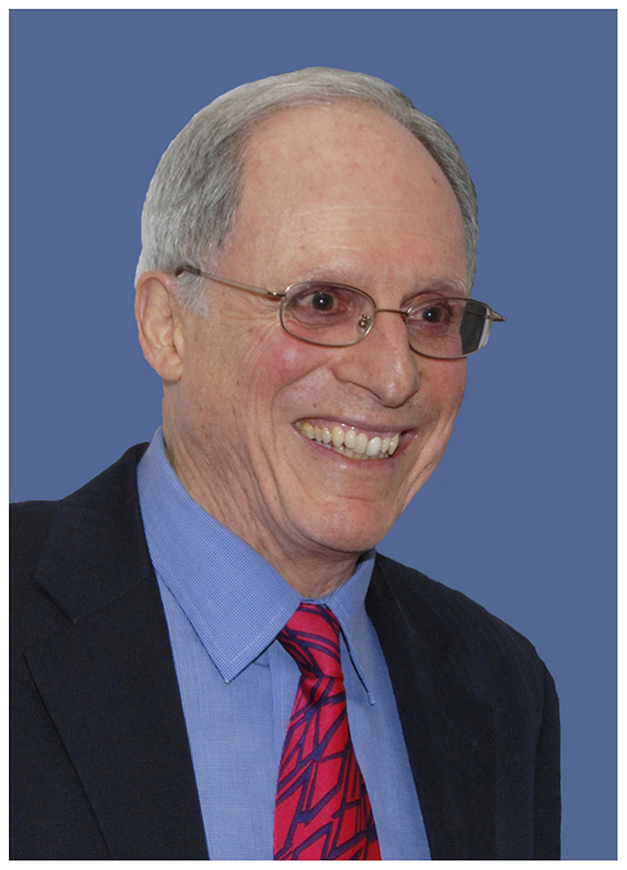
Image 2. David Eisenberg. Photo courtesy of Penny Jennings, University of California, Los Angeles.

## Minoru Kanehisa

Minoru Kanehisa (Image 3) is a Professor in the Institute for Chemical Research at Kyoto University in Japan. Kanehisa received his D.Sc. in physics from the University of Tokyo, and worked at several U.S. institutions, including Los Alamos National Laboratory, where he was a codeveloper of the GenBank database. Kanehisa is one of Japan's most recognized and respected bioinformatics experts. Presently, much of his work focuses on the development and maintenance of the KEGG (Kyoto Encyclopedia of Genes and Genomes) databases, which are used by the global bioinformatics community as tools for understanding and modeling molecular interaction networks and biochemical reactions.

**Figure:**
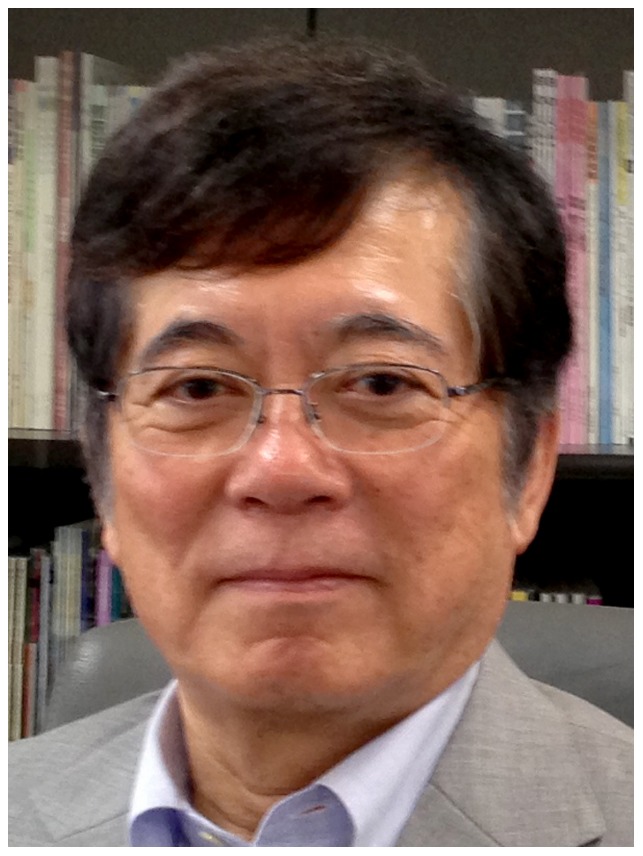
Image 3. Minoru Kanehisa. Image credit: Minoru Kanehisa.

## Satoru Miyano

Satoru Miyano (Image 4) is a Professor at the Human Genome Center, Institute of Medical Science at the University of Tokyo. He holds his Ph.D. in mathematics from Kyushu University in Japan. Miyano is a leader in the computational biology community, is a former member of the ISCB Board of Directors, and has been a key organizer of numerous international bioinformatics and computational biology meetings. He has harnessed the power of supercomputing to develop methods for mining genome and microarray gene expression data sets in order to better define gene networks. His research group has also developed software for modeling and simulating complex biological systems. Since 2006, Miyano's research group has worked on RIKEN's life science grand challenge project titled “Next-Generation Integrated Simulation of Living Matter,” for which they have developed novel petascale computational processes to better merge observational and simulation data.

**Figure:**
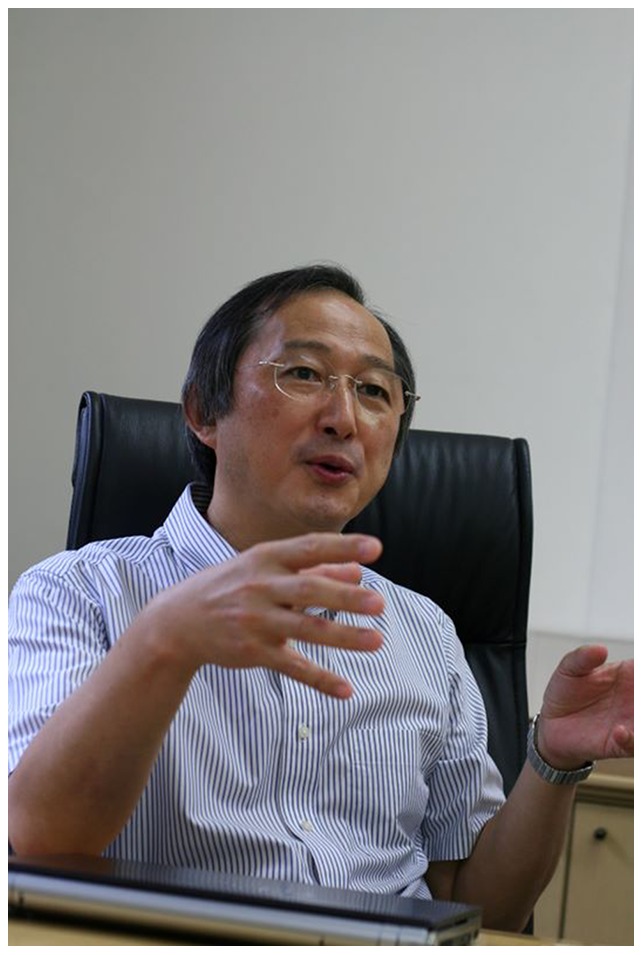
Image 4. Satoru Miyano. Image credit: Satoru Miyano.

## Ruth Nussinov

Ruth Nussinov (Image 5) is a Principal Investigator at the National Cancer Institute of the National Institutes of Health and a Professor in the Department of Human Genetics of the School of Medicine at Tel Aviv University, Israel. She is the Editor-in-Chief of *PLOS Computational Biology* and has served on the editorial boards of several other biomedical journals. Nussinov received her Ph.D. in biochemistry from Rutgers University and is well known for designing the dynamic programming algorithm for predicting RNA secondary structure. She has made significant contributions to the fields of computational biology and biochemistry, particularly through her work in the areas of protein alignment and docking, protein structure and function, and the proposition and development of the conformational selection and population shift model for binding and allostery. Nussinov has authored over 450 scientific papers, and her work is highly cited.

**Figure:**
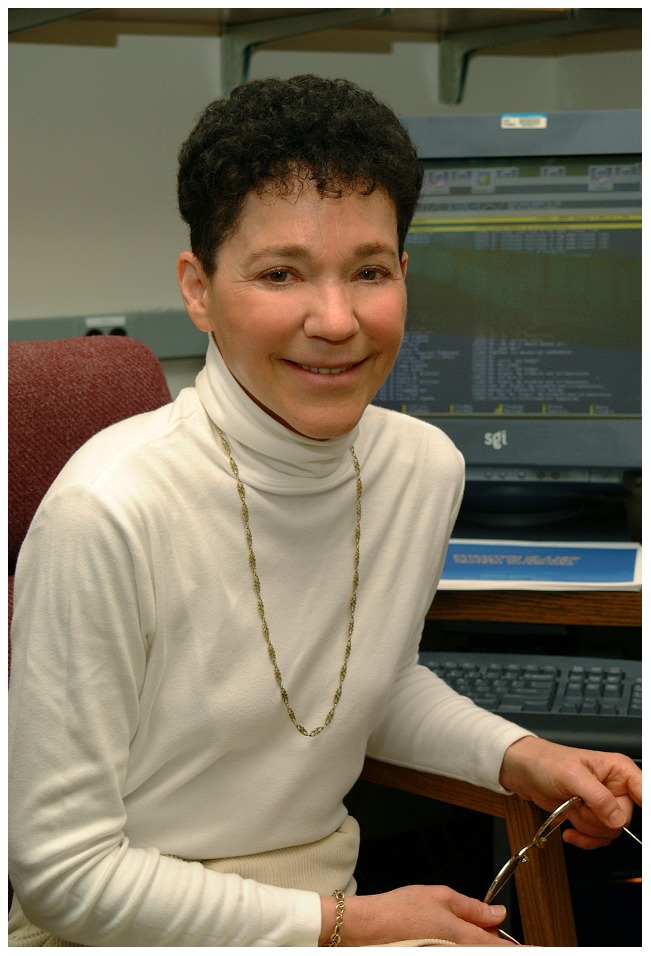
Image 5. Ruth Nussinov. Image credit: Ruth Nussinov.

## Steven Salzberg

Steven Salzberg (Image 6) is a Professor in the Departments of Medicine, Biostatistics, and Computer Science, and Director of the Center for Computational Biology in the McKusick-Nathans Institute of Genetics Medicine, Johns Hopkins University School of Medicine. He is well known for developing scalable algorithms for genomic analysis. Salzberg's group has developed groundbreaking algorithms for next-gen sequence analysis, including open-source software for next-gen sequence alignment, genome assembly, and whole genome alignment. With these tools, Salzberg has been able to explore fundamental biological processes, such as genome evolution and influenza genome dynamics. Salzberg has also had a great impact beyond the scientific community through his advocacy for open-source software and as an author of a popular blog about science and medicine in Forbes.

**Figure:**
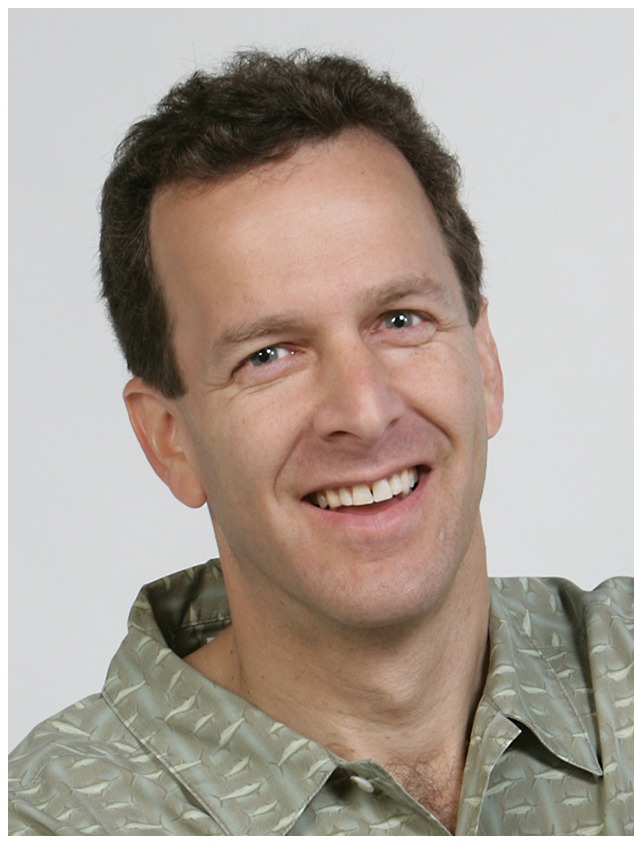
Image 6. Steven Salzberg. Image credit: Steven Salzberg.

